# Comparison of Different Signal Peptides for the Efficient Secretion of the Sweet-Tasting Plant Protein Brazzein in *Pichia pastoris*

**DOI:** 10.3390/life11010046

**Published:** 2021-01-13

**Authors:** Fabrice Neiers, Christine Belloir, Nicolas Poirier, Christian Naumer, Michael Krohn, Loïc Briand

**Affiliations:** 1 Centre des Sciences du Goût et de l’Alimentation, CNRS, INRAE, Université Bourgogne Franche-Comté, F-21000 Dijon, France; fabrice.neiers@u-bourgogne.fr (F.N.); christine.belloir@inrae.fr (C.B.); nicolas.poirier@inrae.fr (N.P.); 2 BRAIN AG, Darmstaedter Str. 34-36, 64673 Zwingenberg, Germany; cn@brain-biotech.com (C.N.); mk@brain-biotech.de (M.K.)

**Keywords:** brazzein, signal peptide, sweet taste receptor, sweet-tasting protein, *Pichia pastoris*

## Abstract

Brazzein is a small sweet-tasting protein found in the red berries of a West African evergreen shrub, *Pentadiplandra brazzeana* Baillon. Brazzein is highly soluble and stable over a large pH range and at high temperatures, which are characteristics that suggest its use as a natural sweetener. However, *Pentadiplandra brazzeana* culture is difficult at a large scale, limiting the natural source of brazzein. Heterologous expression of brazzein has been established in numerous systems, including bacteria, yeast, and transgenic plants. Brazzein requires four disulfide bonds to be active in eliciting an intense sweet taste, and the yeast *Pichia pastoris* appears to be one of the best options for obtaining functional brazzein in high quantities. Employing yeast secretion in the culture medium allows us to obtain fully active brazzein and facilitate purification later. To increase yeast secretion, we compared seven different signal peptides to successfully achieve brazzein secretion using the yeast *P. pastoris*. The brazzein proteins corresponding to these signal peptides elicited activation of the sweet taste receptor functionally expressed in a cellular assay. Among these tested signal peptides, three resulted in the secretion of brazzein at high levels.

## 1. Introduction

Brazzein is a 6.5-kDa sweet-tasting protein containing four disulfide bonds [1,2]. Isolated from the pulp of the berries of a West African plant *Pentadiplandra brazzeana* Baillon, brazzein possesses a sweet taste close to that of sucrose with a high sweetness potency (from 500- to 2000-fold sweeter than sucrose solution on a weight basis). In addition, brazzein presents exceptional thermostability and high solubility over a wide range of pH values, which are essential for food applications [2]. Its long history of human consumption combined with its absence of bitterness makes brazzein a good alternative to natural sweeteners [1]. Moreover, the high water stability, the extreme temperature and pH stability are highly promising for food application [1]. Among the sweet-tasting protein, brazzein appear as one of the most promising. Seven sweet-tasting proteins have been identified to date additionally to brazzein: lysozyme, mabinlin, monellin, neoculin, pentadin and thaumatin.

Natural brazzein has been found to occur in the fruit in two isoforms. The major isoform (54 amino acids, ~80%), called pyrE-bra, contains an N-terminal pyroglutamic acid, whereas the minor isoform (53 amino acids, ~20%), called des-pyrE-bra, lacks the N-terminal pyroglutamate residue [2]. Sensory analyses have revealed that the minor isoform is twice as sweet as the major form [3,4]. The three-dimensional structure of brazzein has been solved by NMR spectroscopy and X-ray crystallography [5,6]. It is composed of three antiparallel beta-strands held together by four disulfide bridges and a short alpha-helix [5,6]. Site-directed mutagenesis experiments combined with cellular assays and sensory analysis have been performed to elucidate the structure-function relationships of brazzein [7,8]. Key amino acid residues responsible for the interaction of brazzein with the TAS1R2/TAS1R3 heterodimeric sweet taste receptor have been identified [7,9]. Although some models of receptor activation by brazzein have been proposed, the exact mechanism is not known. Interestingly, substitutions of some amino acid residues have been shown to significantly increase the sweetness of brazzein [7,10,11].

Because brazzein is difficult to obtain from its natural source, it has been expressed using various recombinant expression systems, including bacteria (*Escherichia coli* and *Lactococcus lactis*) [7,12,13], yeasts (*Saccharomyces cerevisiae, Pichia pastoris* and *Kluyveromyces lactis*) [4,14,15,16] and transgenic plants [17]. The methylotrophic yeast *Pichia pastoris* (also called *Komagataella phaffi*) is known to be safe and is widely used for recombinant protein expression suitable for industrial production. This system allows high-level expression, proper folding, and posttranslational modifications such as phosphorylation and glycosylation under the control of strong and tightly regulated promoters. In addition, proteins can be secreted by the yeast *P. pastoris* at high levels in minimal medium, facilitating purification from the culture supernatant and reducing downstream costs. The natural secretion of proteins is also advantageous in that the protein possesses an N-terminus identical to the natural protein if the signal peptide is properly cleaved. The yeast *P. pastoris* has already been successfully used to express the sweet-tasting proteins thaumatin, monellin, lysozyme and the sweet-taste-suppressing protein gurmarin [18,19,20,21,22,23].

In our previous study, we found that brazzein can be efficiently secreted by the yeast *P. pastoris* under the control of the methanol-inducible alcohol oxidase promoter [4]. Using the yeast prepropeptide signal from the *Saccharomyces cerevisiae* α-mating factor without the Glu-Ala-Glu-Ala spacer peptide, we secreted the two natural isoforms of brazzein, pyrE-bra and des-pyrE-bra, at a level of approximately 90 mg/L. Both recombinant isoforms were purified and biochemically characterized. Mass spectrometry analysis revealed that the signal peptide of both brazzein isoforms was properly cleaved, whereas ^1^H NMR spectroscopy indicated that both proteins were properly folded. In addition, we found that both recombinant brazzein isoforms were functional and able to activate the human sweet taste TAS1R2/TAS1R3 receptor using a cellular assay in agreement with their sensory properties, revealing that these proteins are identical to the brazzein isoforms naturally occurring in the fruit.

An interesting property of yeast *P. pastoris* is its ability to efficiently secrete recombinant proteins in its culture medium. A secreted protein requires the presence of a signal peptide to target the secreted protein to the secretion pathway. The N-terminal part of the mature protein and/or the protein fold are also probably important for an efficient cleaving. For example, the signal peptide cleaving is not occurring properly for lysozyme and thaumatin when expressed in *P. pastoris* [21,22]. It is known that signal peptide selection can influence the amounts of proteins that are secreted in yeast culture medium [24,25]. Unfortunately, incorrect signal peptide processing can occur, generating extra N-terminal residues, which could affect protein functionality [26]. In this study, we compared 6 signal peptides to the previously tested α-factor mating prepropeptide for des-pyrE-bra secretion in *P. pastoris*. The corresponding proteins were purified, characterized and compared for their abilities to activate the sweet taste receptor using a cell-based assay.

## 2. Materials and Methods

### 2.1. Construction of the Expression Vectors

The pPpT4_Alpha_S vector [27] was used for brazzein expression experiments. Synthetic DNA encoding des-pyrE-Bra (mature minor form of brazzein, 53 amino acid residues) fused with the different N-terminal signal peptides was codon optimized for *P. pastoris* expression using GeneDesigner (DNA 2.0, Menlo Park, CA, USA) [28]. The signal peptide cleavage was analyzed using the SignalP 4.0 Server (http://www.cbs.dtu.dk/services/SignalP/). Plasmid names and signal peptide sequences are provided in Table 1. DNA sequences were subcloned in the pPpT4_Alpha_S plasmid between the *EcoRI* and *NotI* restriction sites. *Escherichia coli* strain DH5α was used for amplification of the expression plasmids. The integrity of the constructs was checked by DNA sequencing.

### 2.2. Transformation and Culture Methods

The expression vectors were amplified using the REPLI-g Midi Kit (Qiagen, Hilden, Germany) and linearized with *BamHI* before yeast transformation. The linearized vectors were used for transformation of *P. pastoris* strain CBS7435 [34] via the electroporation method as described in the manual (version 3.0) of the *Pichia* Expression Kit (Invitrogen, Carlsbad, CA, USA). After 48–72 h of incubation at 29 °C on YPDS + zeocin^TM^ (1% *w/v* yeast extract, 2% *w/v* peptone, 1 M sorbitol, 2% *w/v* D-glucose, 400 mg/mL zeocin^TM^), cells were spread on zeocin agar plates. After 72 h, isolated colonies were tested for the Mut^+^ and Mut^S^ phenotypes. For this purpose, transformants were streaked onto minimal dextrose (MD) (2% *w/v* D-glucose, 1.34% *w/v* YNB, 4 mg/mL D-biotin) and minimal methanol (MM) (0.5% *v/v* methanol, 1.34 *w/v* YNB, 4 mg/mL D-biotin) agar plates. Screening of the best secreted clones was performed using mini-culture expression of 10 mL in 50-mL Erlenmeyer shake flasks, as previously described [4]. Having identified the best brazzein-producing transformants for each plasmid construct, large-scale brazzein production was achieved using 3-L shake flasks for 4 days as previously reported [26]. Briefly, isolated transformants were used to inoculate 10 mL of buffered minimal glycerol medium (BMGY: 1% *w/v* yeast extract, 2% *w/v* peptone, 1.34% *w/v* yeast nitrogen base with ammonium sulfate) in a 50-mL baffled flask at 29 °C and 300 rpm for 16 h. Eight milliliters of this preculture was then used to inoculate 800 mL of BMGY divided between two 3-L baffled flasks and incubated at 29 °C and 300 rpm for 24 h. Yeast cells were then harvested by centrifugation (3000× *g*, 5 min), and protein secretion was induced by yeast resuspension in buffered minimal methanol medium (BMM: 1.34% *w/v* YNB, 4 mg/mL D-biotin, 100 mM potassium phosphate, pH 6.0, 1% *v/v* methanol) at 29 °C and 300 rpm for 4 days. During the induction period, methanol was fed every 24 h to maintain a concentration of 0.5% *v/v*.

### 2.3. Purification of Recombinant Brazzein

The yeast supernatant containing secreted brazzein was clarified by centrifugation at 21,600× *g* for 20 min at 4 °C and by filtration (0.22 µm, Startorius, Göttingen, Germany). Brazzein supernatant was dialyzed in three successive steps against 10 L of 50 mM ammonium acetate, pH 4.0, at 4 °C for 2 days and then loaded onto a HiTrap SP-Sepharose column (5 mL, GE Healthcare Biosciences, Uppsala, Sweden) previously equilibrated with 50 mM ammonium acetate buffer, pH 4.0. The column was washed with 50 mM ammonium acetate, pH 4.0, and elution was performed using an increasing NaCl gradient (from 0 to 1 M). A desalting dialysis was operated against 1 L of 20 mM phosphate potassium adjusted to pH 4.0 using phosphoric acid at 4 °C for 1 day. Under magnetic stirring, the pH level of purified brazzein was raised to 7.5 with 1 M potassium phosphate, pH 7.5. Then, under the same conditions, a second dialysis was performed against 50 mM potassium phosphate pH 7.5. Fractions containing brazzein were concentrated using a Vivaspin concentrator (3 kDa molecular mass cutoff), pooled, and stored at −20 °C. The brazzein concentration was determined spectrophotometrically using an extinction coefficient at 280 nm of 8940 M^−1^.cm^−1^.

### 2.4. SDS-PAGE and Mass Spectrometry Analysis

SDS–PAGE (16% acrylamide) was performed using a Mini-Protean II system (Bio-Rad, Hercules, CA, USA). 50 µL of culture medium containing brazzein was precipitated using 450 µL of methanol. After centrifugation, the resulting pellet was loaded on the SDS-PAGE. All gels containing 16% acrylamide were stained using Bio-Safe colloidal Coomassie Brilliant Blue G-250 (Bio-Rad). After a reversed-phase chromatography step, mass spectra were acquired using a Shimadzu 2010EV single quadrupole mass spectrometer in positive ion mode, including profiling. Two microliters of purified brazzein was injected on an Agilent Zorbax 300 SB-C8 column (Agilent, Santa-Clara, CA, USA). The column temperature was set at 30 °C. Then, 0.1% formic acid in water at 0.25 mL/min was applied with a growing gradient to 100% acetonitrile to elute the brazzein.

### 2.5. Functional Characterization of Brazzein Using a Functional Sweet Taste Receptor Assay

Human embryonic kidney cells (HEK293T) stably transfected with chimeric Gα16gust44 protein were cultured in T75 flasks at 37 °C in an incubator kept at 7.3% CO_2_ and 100% air humidity using high-glucose DMEM (Life Technologies, Carlsbad, CA, USA) supplemented with 10% (*v/v*) dialyzed fetal bovine serum (FBS) and 1% penicillin/streptomycin as growth medium [35]. For the calcium mobilization assay, HEK293T-Gα16gust44 cells were seeded at 35,000 cells/100 µL in 96-well black clear plates coated with poly-D-lysine. Twenty-four hours later, using Lipofectamine 2000 (Life Technologies) (0.5 µL per well), cells were transiently transfected with synthetic optimized plasmids coding for cDNA of human TAS1R2 (pcDNA6-TAS1R2, 60 ng per well) and TAS1R3 (pcDNA4-TAS1R3, 60 ng per well) and plasmid pCMV-GCaMP5G (Addgene #31788, 50 ng per well) coding for a green fluorescent protein-based calcium indicator [36,37]. As a negative control, HEK293T-Gα16gust44 cells were mock-transfected with the empty vector. Twenty-four h after transfection, cells were washed with C1 buffer (130 mM NaCl, 5 mM KCl, 10 mM HEPES, 2 mM CaCl_2_, 5 mM sodium pyruvate, pH 7.4) and placed in an automated fluorometric imaging plate reader (Flexstation 3, Molecular Devices, San José, CA, USA). Cells were stimulated with different concentrations of brazzein, and calcium responses were monitored by an increase in fluorescence at 510 nm after excitation at 488 nm. Experiments were performed in duplicate and repeated 4 times. For data analysis, the recorded calcium levels of each well that received the same stimulus were averaged, the response of cells transfected with empty vector (mock cells) was subtracted from that of receptor-transfected cells, and net signals were normalized to background fluorescence (ΔF/F0, F0 fluorescence light before stimulus application). The resulting dose-response data were fitted using a four-parameter logistic equation [f(x) = min + (max − min)/(1 + (x/EC_50_)nH)]. The half-maximal effective concentrations (EC_50_ values) were calculated using SigmaPlot software.

## 3. Results

### 3.1. Construction of Expression Vectors and Transformation into P. pastoris

**Figure 1:**
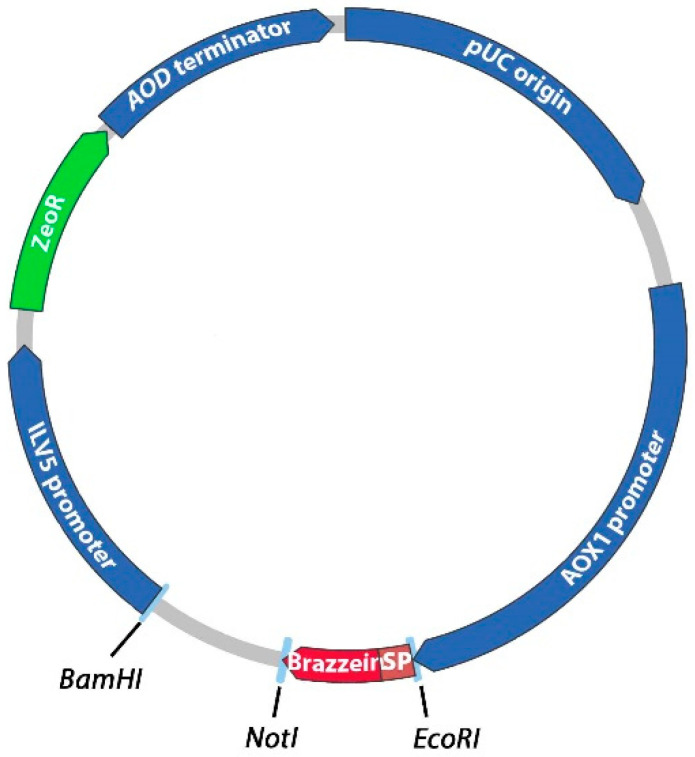
Schematic diagram of the expression plasmids derived from pPpT4_Alpha_S encoding the brazzein fused with the selected signal peptides. The fragment coding mature brazzein and the N-terminal signal peptide (for each different construct) was inserted between the *EcoRI* and *NotI* restriction sites. The sequence coding brazzein plus its signal peptide was expressed under the control of the AOX1 promoter. ZeoR encodes the synthetic resistance factor to zeocinTM (*P. pastoris*/*E. coli* codon optimized) placed under the control of the ILV5 promoter and the AOD terminator. pUC origin codes the *E. coli* replication origin.

To compare the production level and signal peptide processing of brazzein in the yeast *P. pastoris*, we constructed seven different expression plasmids. Brazzein was previously secreted into the buffered minimal medium of *P. pastoris* using the 85-amino-acid secretion signal sequence from the *Saccharomyces cerevisiae* α-mating factor [4]. Since the natural signal peptide of brazzein is not known, the choice to select the best signal peptide (allowing efficient production and secretion) is mainly based on experimental findings. Therefore, we chose to compare the secretion of six other signal peptides with the previously tested α-mating factor (Table 1). The sequence coding the prepropeptide of the α-mating factor was inserted in the same plasmid (corresponding to the pAF-bra plasmid), allowing comparison with the 6 newly tested signal peptide sequences. These signal peptides from various origins were selected because they have been previously successfully used for recombinant protein production and secretion in yeast, as indicated in Table 1. For this purpose, the cDNA sequence encoding mature brazzein fused to the 6 different signal peptide sequences was optimized for yeast expression and synthetized by DNA2.0. The synthetic sequences were subcloned into the *EcoRI* and *NotI* restriction sites of the pPpT4_Alpha_S expression plasmid Figure 1. The resulting expression vectors allow the secretion of brazzein under alcohol oxidase (AOX1) promoter control. Before yeast transformation, the plasmids were digested with *BamHI* to generate integrative expression cassettes. After electroporation of the plasmids in the *P. pastoris* CBS7435 strain, different numbers of transformants were obtained for each construct (approximately 350, 70, 1000, 350, 1000 and 130 clones were obtained for the pAA-, pAE-, pAI-, pAL-, pAS-, and pAV-bra constructs, respectively).

### 3.2. Clones Screening for Brazzein Expression

**Figure 2:**
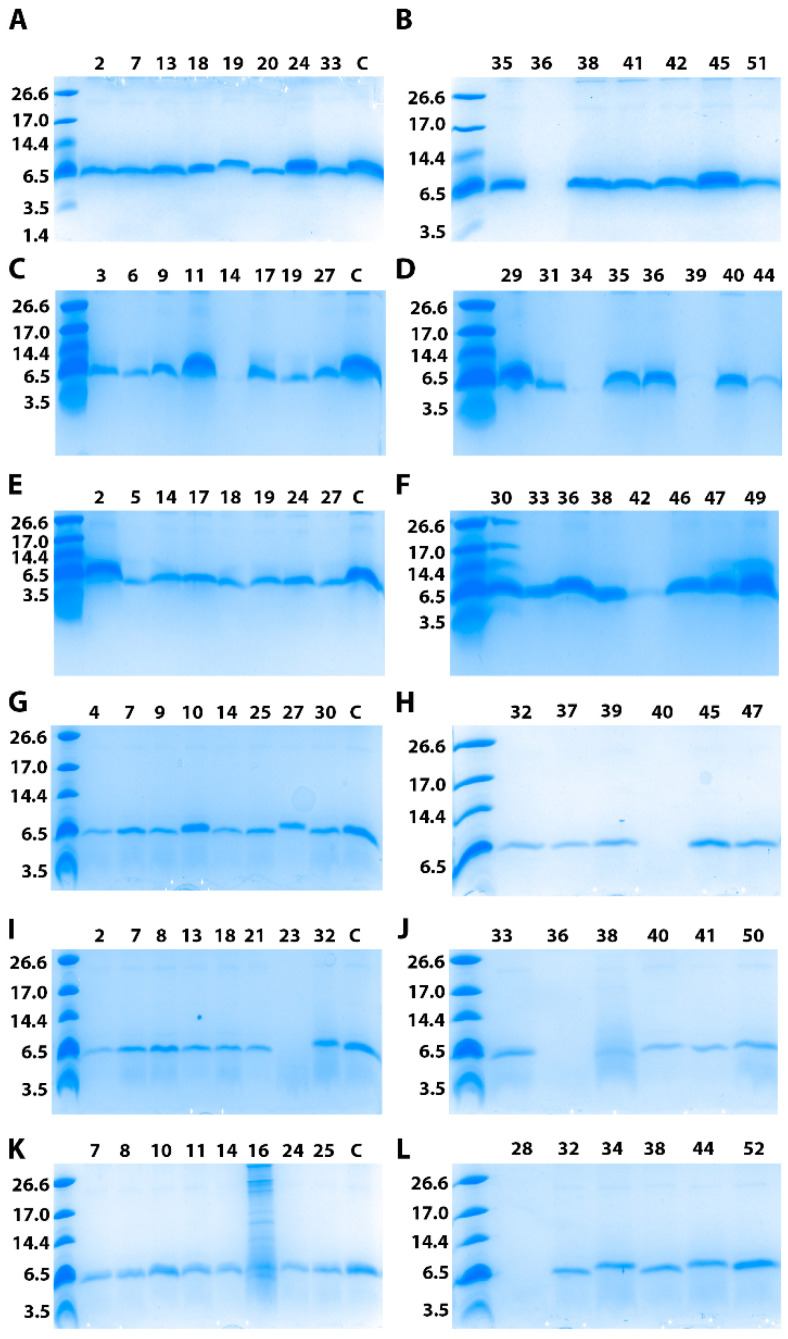
Selection of the *P. pastoris* clone secreting brazzein. SDS-PAGE analysis of 14 or 15 clones selected for each construct among the colonies. (**A**) 10-mL culture was grown for 4 days after induction with methanol before being loaded on a 16% SDS-PAGE gel. The molecular mass standards are indicated on the left in kDa. For each construct, a control (**C**) representing brazzein production under the same conditions as the pAF-bra construct was included as a reference. For each gel, the clone number is also indicated. (**A**,**B**) represent the clones obtained with the pAA-bra construct; (**C**,**D**) the pAE-bra construct; (**E**,**F**) the pAI-bra construct; (**G**,**H**) the pAL-bra construct; (**I**,**J**) the pAS-bra construct; (**K**,**L**) the pAV-bra construct.

For each construct corresponding to the different signal peptides, 52 yeast transformants were isolated and tested on both MD and MM nutritive plates. The Mut^+^ phenotype strains grew well on the two types of plates, allowing characterization of this phenotype compared to the Mut^S^ phenotype presenting limited growth on MM plates. Indeed, the integration of the expression cassette into the AOX1 locus of the *P. pastoris* genome allows either a Mut^S^ phenotype corresponding to a dysfunctional AOX1 gene or a Mut^+^ phenotype corresponding to the functional AOX1 gene. From the 52 tested clones, we found that 36%, 36%, 32%, 32%, 52% and 46% were Mut^S^ phenotypes for the pAA-, pAE-, pAI-, pAL-, pAS-, and pAV-bra constructs, respectively. A set of 15 clones presenting the best growth on plates independent of their phenotype were picked up for 10-mL cultures for 4 days after induction. SDS-PAGE analysis of the yeast supernatants is presented in Figure 2. Interestingly, most of the tested constructs allow secretion of brazzein in the culture medium. Some differences in terms of band intensity can be observed; however, in a small volume, these variations cannot be considered significant. Additionally, for each construct, one clone expressing brazzein derived from the average of the tested clones was selected for larger production and purification, allowing us to compare the amounts of purified protein.

### 3.3. Production and Purification of the Brazzein Secreted with the Different Constructs

**Figure 3:**
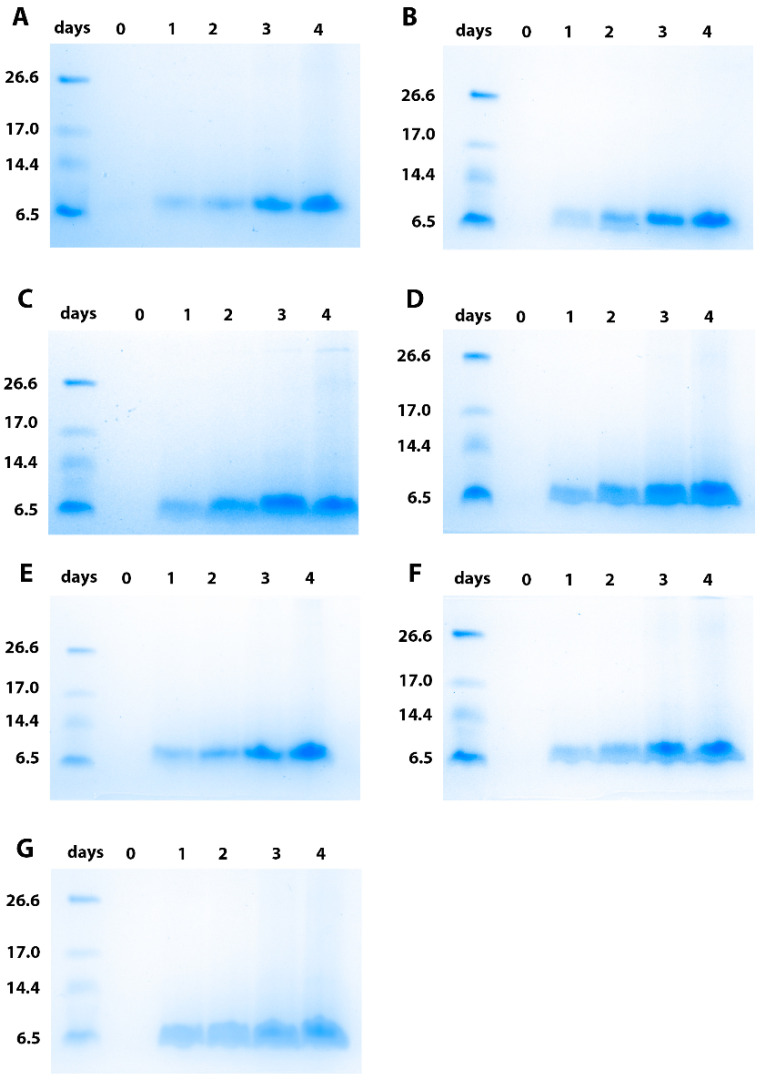
Kinetics of recombinant brazzein secretion by *P. pastoris* using the different constructs in the culture medium, followed by SDS-PAGE analysis. (**A**) shows the strain resulting from the pAF-bra construct as a reference; (**B**) the pAA-bra construct; (**C**) the pAE-bra construct; (**D**) the pAI-bra construct; (**E**) the pAL-bra construct; (**F**) the pAS-bra construct and (**G**) the pAV-bra construct. The molecular mass standards are indicated on the left in kDa.

**Figure 4:**
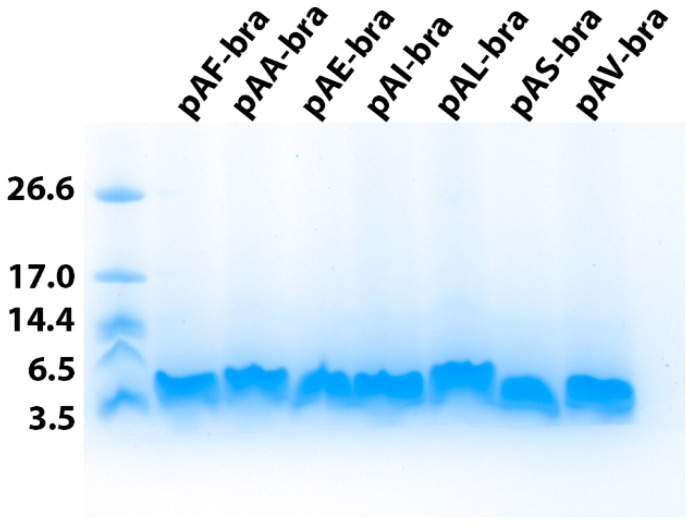
Purified brazzein secreted by the strain expressing the different constructs. SDS-PAGE analysis of brazzein expressed with the 7 different signal peptides. For each purified brazzein, the construct used for production is indicated. The molecular mass standards are indicated on the left in kDa.

One clone resulting from each construct was selected for 400-mL culture using shake flasks. The kinetics of brazzein secretion in the culture medium were analyzed using SDS-PAGE analysis (Figure 3). As already observed previously for the clone resulting from transformation with the pAF-bra construct (secretion with α-mating prepro-factor of *Saccharomyces cerevisiae*), electrophoresis revealed that for all of the constructs, brazzein regularly accumulated in the culture medium during the four days of production. Then, the brazzein secreted using the seven different signal peptides was purified. For this purpose, the sweet-tasting protein was purified from a clarified medium culture using centrifugation and then applied to SP-Sepharose after a dialysis step. Brazzein resulting from the different constructs was eluted at 0.5 mM NaCl during the gradient on SP-Sepharose, suggesting similar physico-chemical properties (data not shown). For each construct, purified brazzein was obtained at a high level of purity after purification, as shown by SDS-PAGE analysis (Figure 4). Similar results were obtained during the two rounds of production and purification, suggesting that the quantity differences are mainly due to the signal peptide. However, it cannot be excluded that the copy number resulting from genomic integration can contribute to this variability. Interestingly, we found that two signal peptides (from α-amylase and lysozyme) allowed us to obtain a quantity of purified brazzein similar to that previously obtained with the *Saccharomyces cerevisiae* α-mating factor (Table 2), whereas the signal peptide of invertase (pAV-bra construct) led to a secretion level 1.5 times lower than that obtained with the pAF-bra vector. In contrast, the signal peptides isolated from Exg1p, serum albumin and inulinase allowed the secretion of brazzein in the culture medium, but at a lower level (between four and seven times lower) than that obtained with the alpha mating factor secretion signal. Mass spectrometry analysis indicated that the six tested signal peptides were properly cleaved during protein export, allowing the production of mature brazzein with the expected mass as well as our control brazzein resulting from the pAF-bra construct (Table 3, Figure S1). However, the three measured masses indicate a mass difference that does not correspond to sequence differences but potentially results from oxidation for two of them and unknown modification associated with the 52 Da lost for the brazzein produced with the invertase signal peptide.

### 3.4. Activation of the Sweet Taste Receptor by Recombinant Brazzein

**Figure 5:**
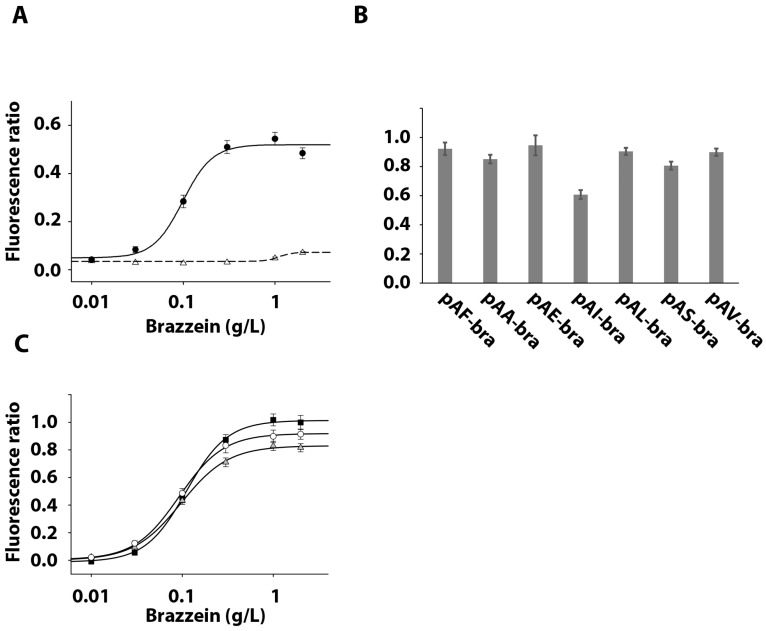
Response of TAS1R2/TAS1R3-expressing HEK293T-Gα16gust44 cells to brazzein produced with the different signal peptides. (**A**) shows the fluorescence ratio measured in TAS1R2/TAS1R3-expressing cells (black circle) and mock-transfected cells (white triangle) with increasing concentrations of brazzein expressed with the *Saccharomyces cerevisiae α*-mating factor. (**B**) presents the calcium responses of TAS1R2/TAS1R3-expressing cells after application of a saturating concentration of 1 g/L brazzein resulting from the different plasmids. (**C**) shows the concentration responses of TAS1R2/TAS1R3-expressing cells with brazzein expressed with the *Saccharomyces cerevisiae* α-mating factor (dark square), the α-amylase peptide signal (gray triangle) and lysozyme (white circle). All the error bars represent standard error means resulting from 2 measurements by experiment and each experiments was repeated 4 times.

To check that the recombinant brazzein produced with the different signal peptides is sweet, we tested its ability to activate the human sweet taste receptor using a cellular-based assay. HEK293T-Gα16gust44 cells [4] were transiently transfected with plasmids coding for the human sweet taste receptor subunits TAS1R2 and TAS1R3 and the GCaMP calcium sensor. Cells were then subjected to calcium imaging experiments. First, the dose-response curve was established by plotting signal amplitude versus log agonist concentration, allowing us to calculate half maximal effective concentrations (EC_50_) for the brazzein secreted using the *Saccharomyces cerevisiae* α-mating factor (Figure 5A). Then, the seven brazzein samples purified at the highest level were tested at a saturating concentration (1 mg/mL) (Figure 5B). For each construct, brazzein elicited a similar calcium signal amplitude, indicating a similar cellular response, confirming that brazzein secreted using the different signal peptides has the same capacity to activate the sweet taste receptor. Moreover, the posttranslational modifications observed on three of the secreted brazzein samples did not modify their ability to stimulate sweet-sensing cells compared to others. Additionally, the dose-response curves for the brazzein obtained with the signal peptide allowing the best expression were measured. They present similar EC_50_ values (0.111 +/− 0.004 g/L, 0.097 +/− 0.004 g/L, 0.092 +/− 0.003 g/L for pAF-bra, pAA-bra and pAL, respectively) suggesting similar sweetness properties (Figure 5C).

## 4. Discussion and Conclusions

By comparing several signal peptides, we found that brazzein production can be slightly increased, offering new alternatives for improving protein secretion in *P. pastoris*. All of the tested signal peptides allow brazzein secretion in the medium with correct processing, demonstrating the compatibility of these sequences for proper processing by the *P. pastoris* secretion machinery. In addition, for all constructs, we observed the ability of purified brazzein to activate the human sweet taste receptor, indicating that the corresponding brazzein is functional and properly folded with the four disulfide bonds formed. Interestingly, we noted differences in efficiencies among the tested signal peptides, supporting their critical role in the protein export step at the level of production. This study conducted on several signal peptides for the same protein provides a unique opportunity for comparison in further studies using the same signal peptides. Compared with the *S. cerevisiae* α-factor prepro-signal, we observed that the chicken lysozyme signal peptide leads to the highest production yield of brazzein. This signal peptide was already shown to be highly effective for the secretion of human lysozyme in *P. pastoris* [32]. However, it cannot be excluded that the production yield ranking between the different signal peptides will change in controlled fermentation compared to the shaking flask culture tested here. Indeed, protein production is usually more efficient using fermenters [38]. In this context, the pAA-bra, pAF-bra and pAL-bra constructs could offer good opportunities to be scaled up for industrial production. *Bacillus licheniformis* alpha amylase [29] and *Saccharomyces cerevisiae* invertase [39] were previously produced using their signal peptides at high levels in the culture medium (up to approximately 2.5 g/L). Generally, the calculated amount of produced protein is directly deduced from the enzymatic activity measured in the culture medium. In our study, we obtained 0.3 g/L brazzein (for the three peptide signals with the best expression) from nonoptimized production that corresponded to purified brazzein, suggesting a good yield compared to these previous studies. This study paves the way to optimize the brazzein production conditions of large-scale industrialization, offering different signal peptides for good secretion in culture medium. In conclusion, brazzein production in the culture medium of *P. pastoris* allows us to obtain a sweet-tasting protein presenting similar characteristics compared to brazzein originating from *Brazzeana pentadiplandra* Baillon but with the advantage of not being limited by resources in the context of tropical forest preservation.

## Figures and Tables

**Table 1:** Sequences of the signal peptides. For each signal peptide, the organism at the origin of the peptide sequence is indicated as well as the protein from which it originates. The amino acid sequences of the signal peptides and the vector name are also indicated.

Construct Name	Signal Peptide	Organism	Sequence: Signal Peptide	References
pAF-bra	α-mating factor	*Saccharomyces cerevisiae*	*	[4]
pAA-bra	α-amylase	*Aspergillus niger*	MVAWWSLFLYGLQVAAPALA	[29]
pAE-bra	Exg1p	*Pichia pastoris*	MNLYLITLLFASLCSA	[30]
pAI-bra	Inulinase	*Kluyveromyces maxianus*	MKFAYSLLLPLAGVSA	[31]
pAL-bra	Lysozyme	*Gallus gallus*	MLGKNDPMCLVLVLLGLTALLGICQG	[32]
pAS-bra	Serum albumin	*Homo sapiens*	MKWVTFISLLFLFSSAYS	[33]
pAV-bra	Invertase	*Saccharomyces cerevisiae*	MLLQAFLFLLAGFAAKISA	[32]

Notes: * MRFPSIFTAVLFAASSALAAPVNTTTEDETAQIPAEAVIGYSDLEGDFDVAVLPFSNSTNNGLLFINTTIASIAAKEEGVSLEKR.

**Table 2:** Amount of purified brazzein for the different signal peptides. Each plasmid construct allowing generation of the different clones (including the selected clone number and its phenotype) is indicated in the first column. The quantities of purified brazzein are indicated in mg of brazzein per liter of cultivated *P. pastoris* from 400 mL of culture.

Construct Name and Clone Used for the Production	Purified Brazzein (mg/Liter of Culture)
pAF-bra #33 Mut^+^	323
pAA-bra #45 Mut^+^	283
pAE-bra #11 Mut^s^	72
pAI-bra #49 Mut^+^	44
pAL-bra #10 Mut^+^	345
pAS-bra #7 Mut^s^	49
pAV-bra #10 Mut^s^	206

**Table 3:** Mass spectrometry analysis. Each plasmid construct allowing generation of the different strains is indicated in the first column. The masses are calculated from the mass spectrometry analysis.

Construct Used for the Production	Measured Mass (Da)	Mass Differences (Da)
pAF-bra	6362 ± 1	0
pAA-bra	6375 ± 1	+13
pAE-bra	6362 ± 1	0
pAI-bra	6361 ± 1	0
pAL-bra	6375 ± 1	+13
pAS-bra	6362 ± 1	0
pAV-bra	6308 ± 1	−52

## Data Availability

The data presented in this study are contained within the article and supplementary material.

## Supplementary Materials

The following are available online at https://www.mdpi.com/2075-1729/11/1/46/s1, Figure S1: Mass spectra analyses of the 6 purified brazzein proteins.
