# Supplementary material for: Comparison of Different Signal Peptides for the Efficient Secretion of the Sweet-Tasting Plant Protein Brazzein in Pichia pastoris
Source: Life (Basel). 2021 Jan 13;11(1):46. doi: 10.3390/life11010046 (PMC7828362; doi:10.3390/life11010046)
Supplement: Supplementary file 1 [file life-11-00046-s001.pdf]

life

Supplementary Materials

Comparison of Different Signal Peptides for the Efficient Secretion of the Sweet-Tasting Plant Protein Brazzein in *Pichia pastoris*

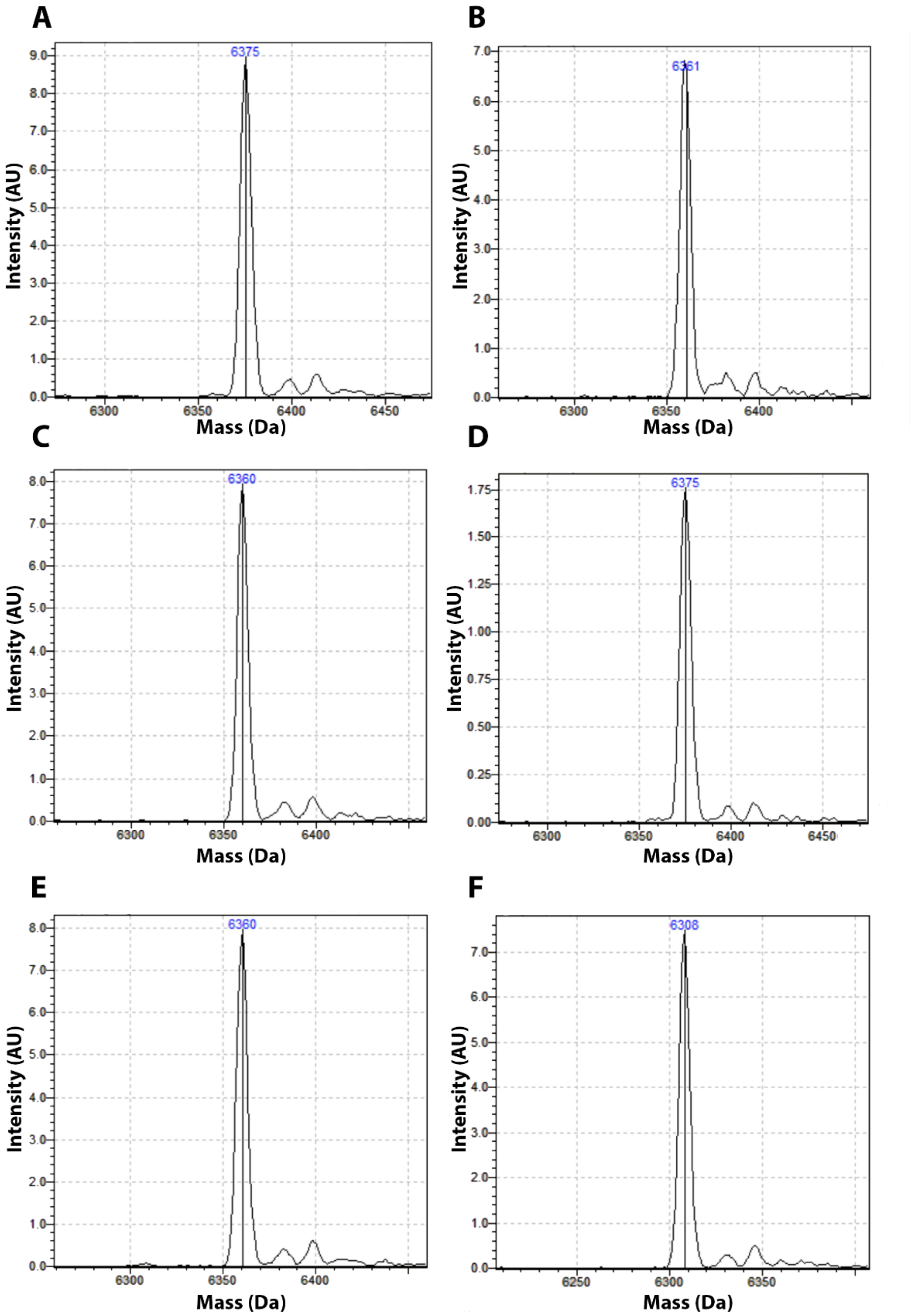

Figure S1. Mass spectra analyses of the 6 purified brazzein proteins. The calculated masses are indicated on the top of the main peak for each brazzein proteins resulting from the pAA-bra (A), pAE-bra (B), pAI-bra (C), pAL-bra (D), pAS-bra (E), pAV-bra (F) constructs.
